# Genome-wide identification of heat shock proteins (Hsps) and Hsp interactors in rice: Hsp70s as a case study

**DOI:** 10.1186/1471-2164-15-344

**Published:** 2014-05-07

**Authors:** Yongfei Wang, Shoukai Lin, Qi Song, Kuan Li, Huan Tao, Jian Huang, Xinhai Chen, Shufu Que, Huaqin He

**Affiliations:** College of Life Sciences, Fujian Agriculture and Forestry University, Fuzhou, 350002 China; Putian University, Putian, Fujian 351100 China

**Keywords:** Rice (*Oryza sativa* L.), Heat shock proteins, Genome wide, Identification

## Abstract

**Background:**

Heat shock proteins (Hsps) perform a fundamental role in protecting plants against abiotic stresses. Although researchers have made great efforts on the functional analysis of individual family members, Hsps have not been fully characterized in rice (*Oryza sativa* L.) and little is known about their interactors.

**Results:**

In this study, we combined orthology-based approach with expression association data to screen rice Hsps for the expression patterns of which strongly correlated with that of heat responsive probe-sets. Twenty-seven Hsp candidates were identified, including 12 small Hsps, six Hsp70s, three Hsp60s, three Hsp90s, and three clpB/Hsp100s. Then, using a combination of interolog and expression profile-based methods, we inferred 430 interactors of Hsp70s in rice, and validated the interactions by co-localization and function-based methods. Subsequent analysis showed 13 interacting domains and 28 target motifs were over-represented in Hsp70s interactors. Twenty-four GO terms of biological processes and five GO terms of molecular functions were enriched in the positive interactors, whose expression levels were positively associated with Hsp70s. Hsp70s interaction network implied that Hsp70s were involved in macromolecular translocation, carbohydrate metabolism, innate immunity, photosystem II repair and regulation of kinase activities.

**Conclusions:**

Twenty-seven Hsps in rice were identified and 430 interactors of Hsp70s were inferred and validated, then the interacting network of Hsp70s was induced and the function of Hsp70s was analyzed. Furthermore, two databases named Rice Heat Shock Proteins (RiceHsps) and Rice Gene Expression Profile (RGEP), and one online tool named Protein-Protein Interaction Predictor (PPIP), were constructed and could be accessed at http://bioinformatics.fafu.edu.cn/.

**Electronic supplementary material:**

The online version of this article (doi:10.1186/1471-2164-15-344) contains supplementary material, which is available to authorized users.

## Background

Plants have evolved a spectrum of molecular programs to adapt to environmental stresses. To survive, plants undergo dramatic changes in physiological and molecular mechanisms [1]. For instance, heat shock proteins (Hsps) are stimulated in response to a wide array of stress conditions and perform a fundamental role in protecting plants against abiotic stresses [1, 2].

Hsps can be classified into five major categories based on molecular mass: small heat shock protein (sHsp) family, chaperonin (Hsp60/GroEL) family, 70-kDa heat shock protein (Hsp70/DnaK) family, Hsp90 family and Hsp100/ClpB family [3]. In *Arabidopsis*, at least 19 genes encoding sHsps, 16 chaperonins, 18 genes encoding Hsp70s, seven Hsp90s, and four Hsp100/ClpBs have been identified through genome-wide analysis [4–9]. Rice is the most important staple food crop in the world and the principal model for other monocotyledonous species [10]. In recent years, researchers have made great efforts on the functional analysis of individual Hsp family members in rice [11–14], however Hsps still have not been fully characterized and little is known about their interactors [14].

Furthermore, detailed studies have established that the overexpression of Hsp70 genes enhanced the plant’s tolerance to environmental stresses [15–17]. Transgenic rice lines that overexpress sHsp17.7 exhibit increased drought tolerance during the seedling stage [18]. However, the cellular mechanisms underlying Hsp function under abiotic stress are not fully understood [3]. The completion of the Rice Genome Sequencing Project and high-throughput experimental methods have generated valuable data that can be used to identify proteins that interact with Hsps in rice, and consequently decipher the functions of Hsps.

Many computational approaches have been proposed to predict protein-protein interactions. In terms of test dataset types, these approaches can be grouped into three classes: sequence-oriented methods [19–22], gene expression profile-based methods [23] and structure-oriented methods [24]. Interolog, a sequence-oriented method, has been widely used to construct protein-protein interactions (PPIs) in diverse organisms [10, 25–27]. This method is based on the principle that orthologous pairs can be detected by mapping those known interactions in the source organism onto the target organism [21]. The gene expression profile-based methods identify genes that exhibit correlated changes in expression over conditions, since they tend to have similar functions or be involved in cellular processes [23, 28]. Each protein interaction mapping technique has different advantages and disadvantages [29], and the techniques are complementary to some extent. In this study, we integrated interolog- and gene expression profile-based methods to identify the interactors of Hsps in rice.

To carry out more reliable functional analysis, we first conducted a genome-wide screening for the true Hsps in rice using integration of orthology and expression association data. Then, we used interolog- and expression profile-based methods to identify Hsp70s interactors in rice response to abiotic stresses. Through mining the signal behind their interactors, we further investigated the pattern of binding sites and the interaction network of Hsp70s in response to abiotic stresses.

## Results

### Gene expression in rice subjected to abiotic stresses

**Figure 1:**
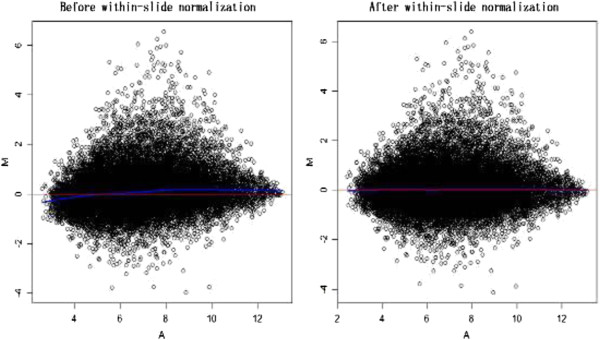
**Within-slide normalization of rice GeneChips.** *M* was the log intensity ratio and *A* was the average log intensity for a dot in the plot. Each point represented the expression pattern of a probe-set in the plot. The horizontal red lines represented the theoretical median of the global *M*-values. The continuous blue curves indicated the global trend line, as estimated by LOWESS regression. (Left) *MA*-plot before within-slide normalization; (Right) *MA*-plot after within-slide normalization.

**Figure 2:**
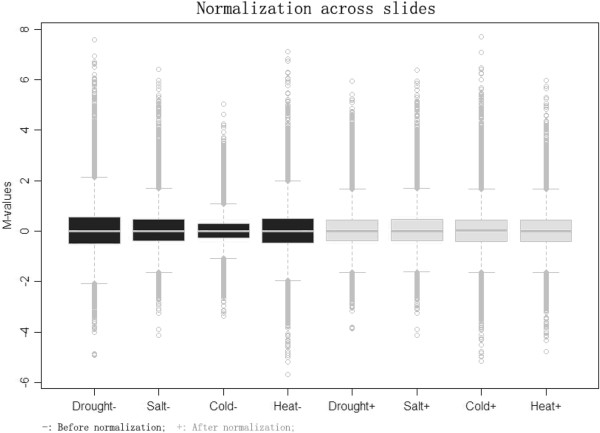
**Multiple-slide normalization among rice GeneChips.** Black boxplots (left) showed the spread of *M*-values in four kinds of GeneChips before multiple-slide normalization. The array for cold treatment had a much narrower spread compared with the others. Gray boxplots (right) represented the spread of *M*-values in the same four arrays after multiple-slide normalization.

Four sets of gene expression data from rice seedlings exposed to drought, salt, cold and heat treatment were collected (Table 1) from the Gene Expression Omnibus (GEO) [30]. The K-nearest neighbor (KNN) impute method was used to estimate the missing values in GeneChips [31]. A total of 22,707 probe-sets with detectable expression values were selected from these GeneChips. Within-slide normalization (Figure 1) and multiple-slide normalization (Figure 2) were performed sequentially to minimize systematic variations.

**Table 1:** **Rice GeneChips in response to abiotic stresses**

Stress	Drought	Salt	Cold	Heat
ID	GSE6901	GSE6901	GSE6901	GSE14275
Platform	GPL2025	GPL2025	GPL2025	GPL2025
Organism	*Oryza Sativa*	*Oryza Sativa*	*Oryza Sativa*	*Oryza Sativa*
Sample	Seedling	Seedling	Seedling	Seedling
Stress/Control	3/3	3/3	3/3	3/3

**Figure 3:**
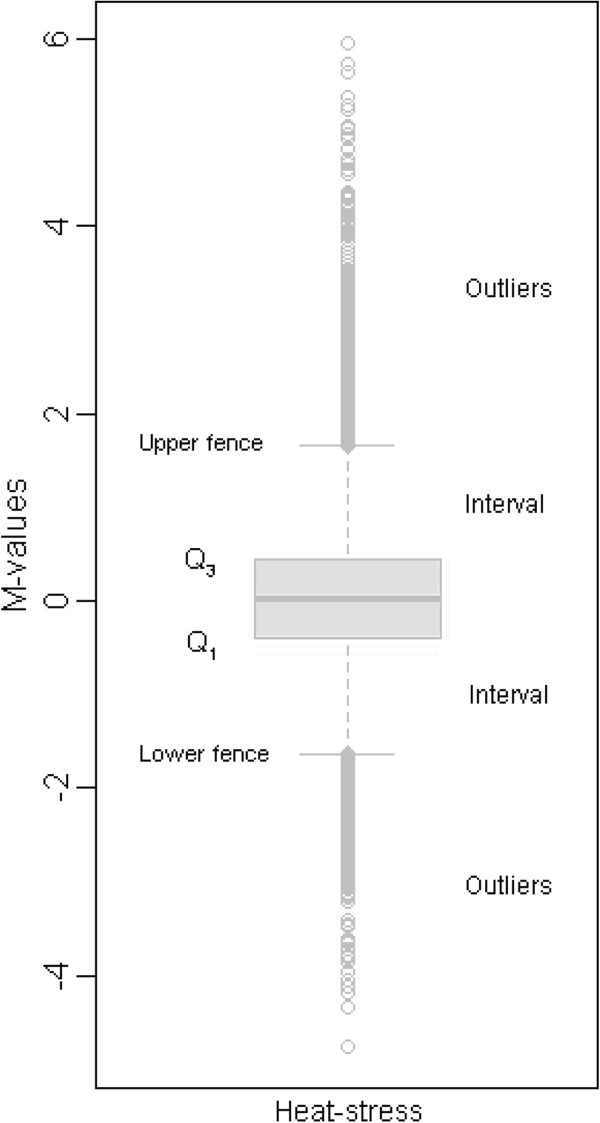
**Boxplot of M-values in response to heat stress.** Q_1_ (−0.392) and Q_3_ (0.432) represented the lower quartile and the upper quartile, respectively. The interval equaled 1.5× the interquartile range (IQR). The upper fence lay at Q_3_ + 1.5×IQR (1.668), while the lower fence lay at Q_1_-1.5×IQR (−1.628). The outliers represented observations that fell beyond the upper and lower fences.

**Figure 4:**
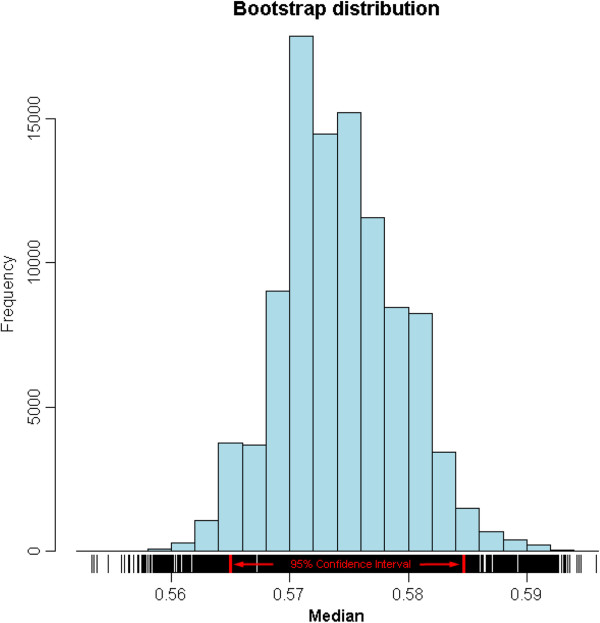
**Bootstrap distribution of the estimated median absolute PCC value between the expression value of any two probe-sets in the GeneChips.** Ten thousand non-redundant probe pairs were randomly selected, and the absolute PCC value between each pair was computed. Based on these 10,000 PCC values, 100,000 bootstrap samples were built by sampling with replacement, and the 95% confidence interval of the global median absolute PCC value was determined as ranging from 0.5648 to 0.5842.

Then, we identified heat-responsive (HR) probe-sets and estimated the global gene-gene pairwise relationships. In this study, we applied boxplots [32, 33] to identify HR probe-sets, which were defined as a group of probe-sets that were significantly up- or down-regulated by heat treatments. A total of 1,135 (5%) HR probe-sets that were expressed differentially under heat stress were detected (Figure 3). Among them, 651 probe-sets were up-regulated, while 484 probe-sets were down-regulated. Meanwhile, bootstrap analysis [34] was performed to estimate the absolute median value of Pearson Correlation Coefficients (PCC) between any pair of genes. The bootstrapped 95% confidence interval for the population ranged from 0.5648 to 0.5842 (Figure 4).

### Genome-wide identification of Hsps in rice

Hsps screening in the rice proteome consisted of three steps. First, 41 candidate protein sequences, which were annotated as Hsps and contained the characteristic domains (Additional file 1: Table S1) of Hsps in Uniprot database [35], were downloaded. These sequences included 23 small Hsps (sHsps), eight Hsp70s, four Hsp60s, three Hsp90s and three Hsp100/ClpBs. Second, 10 of the 41 candidate proteins, whose expression value was absent in GSE6901 (GeneChips for drought, salt, and cold treatments) or GSE14275 (GeneChip for heat treatment), were filtered out. Third, since Hsps can stimulate a wide range of HR genes [3, 36], and those genes involved in similar functions or cellular processes are likely to have similar expression profiles over conditions [23]. So we supposed the true Hsp genes should have a higher expression correlation with HR probe-sets compared with other genes. Therefore, 27 candidate genes, whose expression patterns were similar to that of the HR probe-sets (Table 2), were ultimately recognized as Hsps, including 12 sHsps, six Hsp70s, three Hsp60s, three Hsp90s and three Hsp100/ClpBs (Table 3). The average absolute value of the PCC between them and HR probe-sets reached 0.76, which was markedly greater than that of the global pairwise values (0.5648-0.5842) and the value of the Ubq5/control (0.5089).

**Table 2:** **PCC between Hsps and heat responsive probe-sets in rice in response to abiotic stresses**

Uniprot	MSU-ID	Family	|PCC| with UP*	|PCC| with DP*	Average
Q6Z7B0	LOC_Os02g02410	Hsp70	0.8035	0.8622	0.8328
Q75GT3	LOC_Os03g31300	Hsp100/ClpB	0.8019	0.8614	0.8316
Q943E6	LOC_Os01g04380	sHsp	0.8016	0.8546	0.8281
Q10SR3	LOC_Os03g02260	Hsp70	0.7914	0.8579	0.8246
Q6K7E9	LOC_Os02g54140	sHsp	0.7871	0.8610	0.8241
Q0E3C8	LOC_Os02g08490	Hsp100/ClpB	0.7817	0.8551	0.8184
Q84J50	LOC_Os03g16040	sHsp	0.7956	0.8404	0.8180
Q10PW8	LOC_Os03g11910	Hsp70	0.7956	0.8401	0.8179
Q5Z9N8	LOC_Os06g50300	Hsp90	0.7755	0.8410	0.8082
Q6F2Y7	LOC_Os05g44340	Hsp100/ClpB	0.7590	0.8367	0.7978
Q8H903	LOC_Os10g32550	Hsp60	0.7810	0.8117	0.7963
P27777	LOC_Os01g04370	sHsp	0.7770	0.8101	0.7936
Q0E4A8	LOC_Os02g03570	sHsp	0.7541	0.8209	0.7875
Q67X83	LOC_Os06g11610	sHsp	0.7316	0.8028	0.7672
B7EZJ7	LOC_Os02g10710	sHsp	0.7341	0.7828	0.7585
Q6Z7V2	LOC_Os02g52150	sHsp	0.7264	0.7902	0.7583
Q9AQZ5	LOC_Os01g08560	Hsp70	0.7181	0.7938	0.7560
Q2QV45	LOC_Os12g14070	Hsp70	0.7471	0.7504	0.7488
Q84Q72	LOC_Os03g16030	sHsp	0.7313	0.7655	0.7484
Q10RW9	LOC_Os03g04970	Hsp60	0.7375	0.7393	0.7384
Q9LWT6	LOC_Os06g02380	Hsp60	0.7329	0.7338	0.7333
Q84Q77	LOC_Os03g15960	sHsp	0.6815	0.7351	0.7083
Q943K7	LOC_Os05g38530	Hsp70	0.6785	0.7363	0.7074
P31673	LOC_Os03g16020	sHsp	0.6464	0.6942	0.6703
Q0J4P2	LOC_Os08g39140	Hsp90	0.6045	0.6558	0.6301
Q7EZ57	LOC_Os07g33350	sHsp	0.6393	0.5777	0.6085
Q69QQ6	LOC_Os09g30418	Hsp90	0.5857	0.6165	0.6011
**\**	**Global |PCC|**	CI_upper**	**\**	**\**	**0.5842**
**\**	**Global |PCC|**	CI_lower**	**\**	**\**	**0.5648**
**P0C031**	**LOC_Os06g44080**	**Ubq5/control**	**0.5547**	**0.4631**	**0.5089**
Q943E9	LOC_Os01g04350	sHsp	0.4685	0.5228	0.4957
Q7X9A7	LOC_Os03g64210	Hsp60	0.5258	0.4232	0.4745
Q6AUW3	LOC_Os05g42120	sHsp	0.4727	0.3719	0.4223
Q10NA9	LOC_Os03g16860	Hsp70	0.4162	0.3292	0.3727

*UP: Probe-sets that were significantly up-regulated by heat treatments; DP: Probe-sets that were significantly down-regulated by heat treatments.

**CI_upper: upper bound of bootstrapped 95% confidence interval for global pairwise |PCC|; CI_lower: lower bound of bootstrapped 95% confidence interval.

Controls shown in BOLD.

**Table 3:** **Numbers of Hsps identified in this paper**

Families	sHsp	Hsp60	Hsp70	Hsp90	Hsp100	Total
First step	23	4	8	3	3	41
Second step	14	4	7	3	3	31
Third step	12	3	6	3	3	27

First step: Proteins that were annotated as heat shock proteins and contained the specific domains of heat shock proteins were downloaded from Uniprot database; Second step: Hsp candidates, whose expression value was absent in GSE6901 or GSE14275, were filtered out; Third step: Candidates, whose expression patterns were strongly correlated with the patterns of the HR probe-sets, were ultimately recognized as heat shock proteins.

### Genome-wide identification of the interactors of Hsps in rice, with a focus on Hsp70s

Using the interolog method, 9,132 potential PPIs related to Hsps in rice (Additional file 1: Table S3) were mapped from the experimentally identified PPI in yeast [37]. The predicted PPIs corresponding to 6 Hsp70s accounted for nearly 45% of the total interactions (4,091 out of 9,132). Therefore, in this paper, Hsp70s were selected as a case study.

Each of 6 Hsp70s sequences was used as a query to search its interactors in rice based on interlog method. After that, we applied an expression profile-based method to reduce the false-positive rate of Hsp70s PPIs predicted by interolog. The expression relationship between each interacting partner was further measured by Pearson Correlation Coefficients (PCCs). We found that the absolute PCC of 1,072 PPIs related to Hsp70s, including 430 interactors, were greater than 0.90 (Additional file 2: Supplemental Data 1A). Upon exposure to abiotic stresses, the expression of 166 interactors showed a positive relationship with that of Hsp70s, while the expression of 264 interactors was negatively correlated with that of Hsp70s (Table 4).

**Table 4:** **Number of Hsp70s interactors predicted by Interolog and co-expression methods**

+/− correlated with Hsp70s	Interactors	Interaction
Positively correlated	166	393
Negatively correlated	264	679
Total	430	1072

### Assessment of the PPIs of Hsp70s in rice

Two computational methods were used to evaluate the overall quality of the above prediction. Randomized PPIs were generated and used as a control.

**Figure 5:**
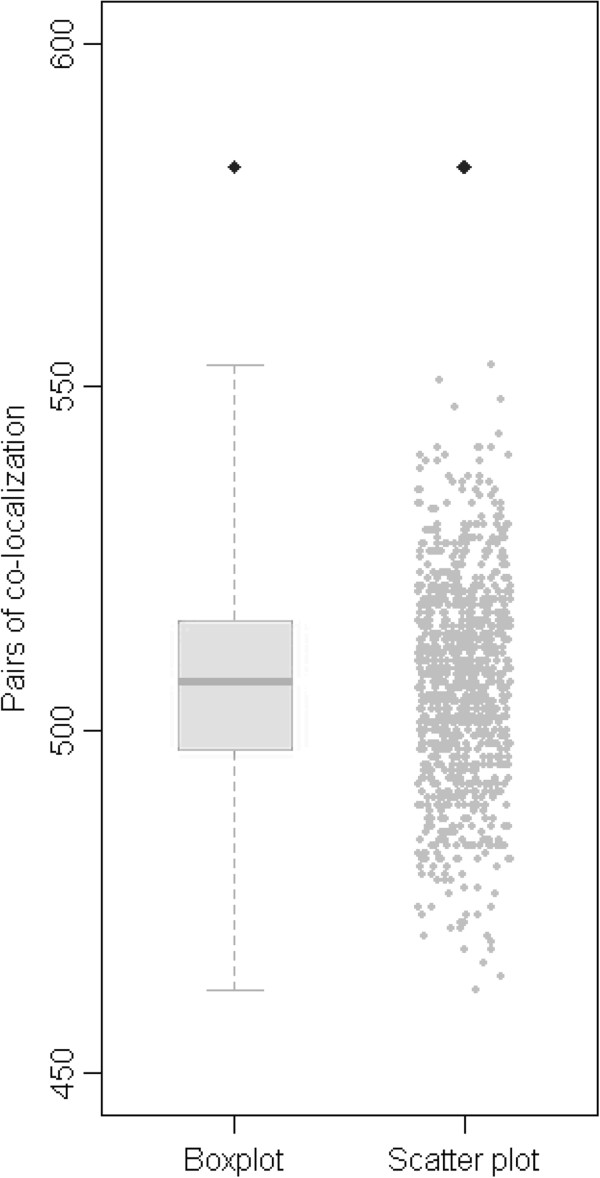
**Number of predicted interaction pairs localized in the same subcellular organelle.** Black dots showed the number of pairs localized to a common cellular compartment in the predicted PPIs. Boxplot and scatter plots represented the distribution of the number in 1,000 randomly repeated PPIs.

First, the co-localization method was applied to assess the Hsp70 PPIs. This method is based on the principle that interacting proteins are more likely to localize to the same cellular compartment than randomized pairs [38]. The subcellular localization annotation of each protein in rice was obtained from WoLF PSORT [39], a stringent protein localization predictor based on experimental data. All of the predicted Hsp70s interactors contained subcellular localization annotations (Additional file 2: Supplemental Data 1B). We found that 582 PPIs (54% of 1,072 predicted PPIs) localized in common cellular compartments. In contrast, the maximum number of PPIs localized in the same subcellular compartment in 1,000 randomly repeated networks was 553 (51% of 1,072 randomized PPIs) (Figure 5), which was significantly lower than that of the predicted Hsp70 PPIs (empirical *p*-value < 0.001).

**Figure 6:**
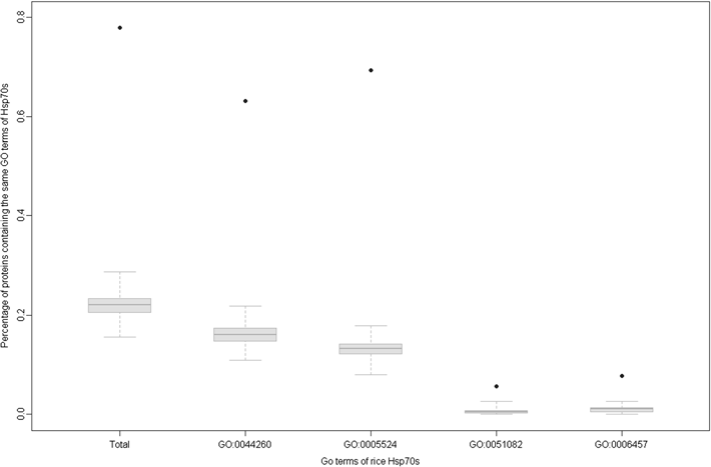
**Percentage of interactors that had the same GO annotation as Hsp70s.** Black dots represented the percentage of predicted interactors that shared the same GO annotations as Hsp70s. The boxplot showed the distribution of that in 1,000 randomized repeats of Hsp70s interactors.

Second, we used the co-function method to test the overall quality of predicted Hsp70s PPIs. This method is based on the assumption that interacting partners tend to participate in the same cellular processes or share similar functions [22, 39]. The 6 Hsp70s contained four different GO terms (GO:0044260, GO:0005524, GO:0051082 and GO:0006457) in biological processes (BPs) or molecular functions (MFs). The result showed that 385 of 430 predicted Hsp70 interactors had GO annotations (Additional file 2: Supplemental Data 1B), and 300 of these interactors (78%) shared at least one common GO term with Hsp70s. The proportion of predicted interactors sharing the term GO:0044260, GO:0005524, GO:0051082 and GO:0006457 were 243 (63%), 267 (69%), 22 (6%) and 30 (8%), respectively, significantly higher than that of 1,000 repeats of randomized Hsp70 interactors (empirical *p*-value < 0.001) (Figure 6).

### Identification of the binding sites of Hsp70s in rice

The above assessments provided strong support for the reliability of the Hsp70 interactors predicted in this paper. Therefore, we used these interactors as the positive dataset, and constructed a negative dataset composed of 10,158 proteins that were less likely to interact with Hsp70s. Since binding sites tend to occur more frequently in interacting proteins than in non-interacting proteins [40], we sought to detect over-represented domains or motifs by comparing their frequency of occurrence in the two different datasets.

The annotations of rice protein domains were obtained from Pfam [41]. We identified 102 domains of 397 proteins in the positive dataset (Additional file 2: Supplemental Data 1B), and 2,628 domains of 7,746 proteins in the negative dataset. The number of negative samples was much greater than that of positive samples (20:1). To reduce this bias, we implemented one-tailed Fisher’s exact test [42] to detect the over-represented domains in the coordinated datasets (i.e., 397 positive samples versus 794 samples in the negative dataset; a ratio of 1:2), and used the Benjamini and Hochberg (BH) method [43] to control the false discovery rate (FDR). In addition, the above procedure was repeated 10 times by randomly changing the negative samples. Finally, 13 domains were detected with *p*-value lower than 0.05 in the 10 replicas (Additional file 3: Supplemental Data 2A). Similarly, we analyzed the binding motifs of Hsp70s in rice. The motif annotations were acquired from PROSITE [44, 45]. There were 113 motifs in 404 proteins among the positive samples (Additional file 2: Supplemental Data 1B), while there were 1,071 motifs in 10,081 proteins among the negative samples. Twenty-eight overrepresented motifs were ultimately investigated (Additional file 3: Supplemental Data 2B).

### Functional analysis of Hsp70s in rice

It is expected that the functions of proteins can be deduced from their interactors. As mentioned above, among the 430 interactors of Hsp70s, 385 have BP or MF GO annotations (Additional file 2: Supplemental Data 1B). Furthermore, 147 interactors, whose expression levels positively correlated with that of Hsp70s, contained 109 GO annotations. In contrast, the 238 interactors, whose expression levels negatively correlated with Hsp70s, had 90 different GO annotations. The two distinct groups were defined as Positively Correlated Interactors (PCIs) and Negatively Correlated Interactors (NCIs). Using GO enrichment analysis, we found that 24 BP GO terms and five MF GO terms with *p*-values less than 0.05, were enriched in the PCIs compared with that in NCIs (Additional file 4: Supplemental Data 3A), suggesting that these biological processes or functions would be induced with the up-regulation of Hsp70s. Meanwhile, 23 BP GO terms and 16 MF GO terms with *p*-values less than 0.05 were over-represented in the NCIs compared with that in the PCIs (Additional file 4: Supplemental Data 3B), indicating that these biological processes or functions would be induced as Hsp70s down-regulation.

### Construction of tools and riceHsp database

**Figure 7:**
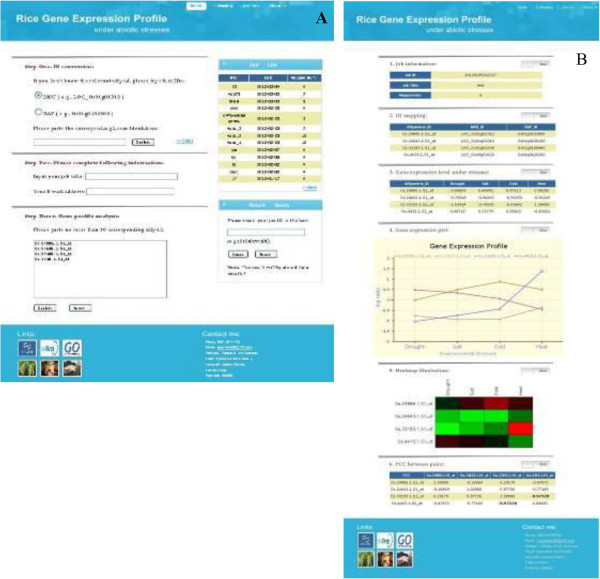
**Screenshot of the RGEP database. (A)** The RGEP homepage. **(B)** Sample search result provided by RGEP.

**Figure 8:**
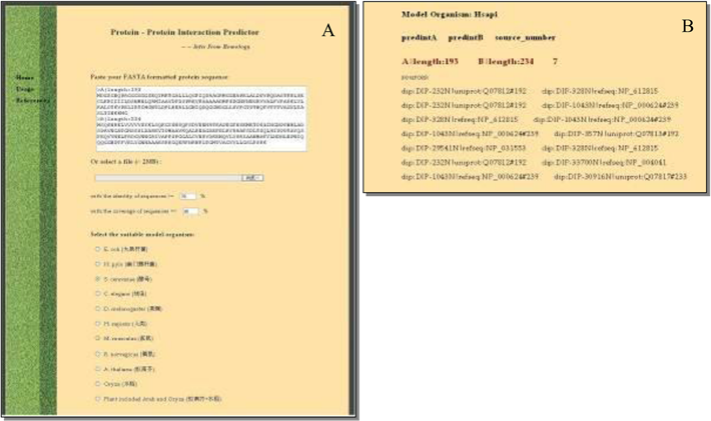
**Screenshot of the PPIP website. (A)** PPIP homepage. **(B)** The predicted result provided by PPIP.

We constructed two databases, named Rice Heat Shock Proteins (RiceHsps) and Rice Gene Expression Profile (RGEP), and one online tool, named Protein-Protein Interaction Predictor (PPIP). The RiceHsps was built to store and show our predicted results in this paper. The RGEP was constructed to store the integrated gene expression data for rice subjected to abiotic stresses, including drought, salt, cold and high temperature. It also provided a function for identifier conversion among *Michigan State University Osa1 Rice Locus* (MSU ID), *Rice Annotation Project Locus* (RAP ID) and *Affymetrix Rice Genome Probe-set* (Affymetrix ID) (Figure 7). The tool PPIP was developed based on the interolog method. Once the user uploads at least two protein sequences in FASTA format into the text area, or a sequence file less than 2 Mb, the corresponding orthologous protein pairs, whose interaction has been verified by biochemical experiments in the selected model organism, will be retrieved (Figure 8). These online databases and tool can be accessible at http://bioinformatics.fafu.edu.cn.

## Discussion

### Heat shock proteins (Hsps) in rice

Using a combination of orthology and expression association data, we identified 27 heat shock proteins, including 12 sHsps, 6 Hsp70s, 3 Hsp60s, 3 Hsp90s and 3 Hsp100/ClpBs. Using an orthology-based strategy, Sarkar et al. (2009) identified 23 sHsps in rice [11], 12 of which were confirmed in this paper and showed a strong relationship with HR probe-sets under abiotic stresses. According to orthology- and expression level-based data, Singh et al. (2010) discovered three Hsp100/ClpB proteins in rice [12], which were consistent with the result of this paper. We further noted that the expression pattern of the three Hsp100/ClpBs closely resembled that of HR probe-sets under abiotic stresses. Recently, Sarkar et al. (2013) identified 32 Hsp70 genes through sequence analysis and orthology-based method [13], including all the six Hsp70s in this paper. However, in this study, we not only adopted the sequence and orthology information, but also the gene expression association information to identify true Hsps in rice. Given that similar proteins in different species may have different functions, one has to take into account that an orthology-based strategy alone is not adequate to identify true Hsps in rice. Furthermore, it is not reliable to screen Hsps for evaluating the gene expression levels of candidates in rice in response to high-temperature stress, because some Hsps express constitutively [3]. Therefore, we used a combination of orthology and expression association data to identify a highly reliable Hsps in rice.

### Binding sites of Hsp70s in rice

Investigating the binding sites of Hsp70s will provide insight into the activity of those proteins and improve our ability to predict the potential risks of a particular mutation. In this study, we identified 13 domains and 28 motifs that occurred more frequently in the positive dataset than in the negative dataset, suggesting that these sequences are potential target sites for Hsp70s in rice. The results were partially supported by biochemical experiments conducted in previous studies. For instance, our results showed that the J-domain (PF00226, PS50076) of DnaJ/Hsp40 was the binding site for DnaK/Hsp70. By point mutation analysis, Wall et al. (1994) demonstrated that the J-domain interacted with DnaK and regulated DnaK activity [46]. Suh et al. (1998) found that the ATPase domain of DnaK was a binding pocket for the J-domain [47]. Horne et al. (2010) suggested that the fusion of the J-domain with p5 (Jdp5) could dramatically stimulate ATP hydrolysis by DnaK, and NMR studies on Jdp5 further indicated that the peptide tethered the J-domain to the ATPase domain of DnaK [48].

Therefore, the results of this study provided useful clues for experimental biologists in further analyzing the function of Hsp70s.

### The Hsp70 interaction network in rice

**Figure 9:**
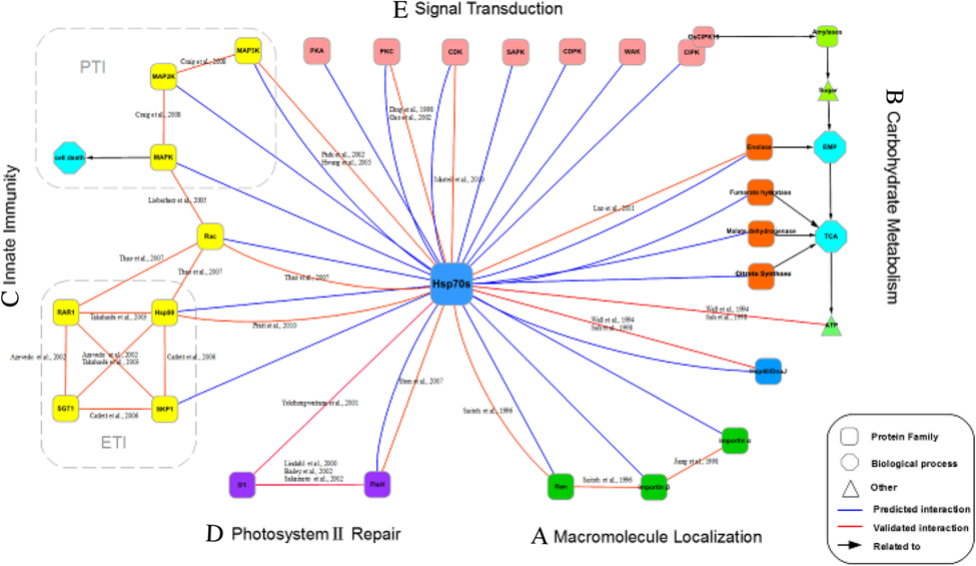
**PPI network of Hsp70s in rice. (A)** Sub-network A: Macromolecule localization. **(B)** Sub-network B: Carbohydrate metabolism. **(C)** Sub-network C: Innate Immunity. ETI, effector - triggered immunity process; PTI, PAMP-triggered immunity process. **(D)** Sub-network D: Photosystem II repair. **(E)** Sub-network E: Protein kinase activities. Red curves indicated known and published interactions, whereas blue curves indicated potential interactions detected in this paper.

The Hsp70s network was shown in Figure 9, and described in the following sections. We classified the interaction network into five sub-networks.

### Sub-network A: Macromolecular translocation

Our results showed that the small GTPase Ran (LOC_Os01g42530), importin α (LOC_Os01g14950, LOC_Os05g06350) and importin β (LOC_Os05g28510) could bind to Hsp70s. Hsp70 and importin β were previously identified as Ran-interacting proteins (Rips) [49]. The results of this study indicated that the Ras family domain (PF00071) and ATP/GTP-binding site motif A (P-loop) (PS00017) of the small GTPase Ran were potential interacting sites of Hsp70s. Furthermore, the expression of Ran and importin proteins was strongly correlated with that of Hsp70s (PCC > 0.90) under abiotic stresses (Additional file 5: Figure S1; Additional file 1: Table S5). We then constructed a protein-protein interaction network consisting of Hsp70s, GTPase Ran and importin proteins in rice (Figure 9A).

Importin α recognizes the nuclear localization signal (NLS) of nuclear proteins in the cytoplasm, forming a stable complex termed the nuclear pore-targeting complex (PTAC) [50, 51]. Importin β docks the PTAC to the cytoplasmic face of the nuclear pore complex (NPC) [52], a channel for macromolecules into the nucleus [53]. In addition, the hydrolysis of GTP by the small GTPase Ran has been shown to be essential for the translocation of docked PATC into the nucleus [54]. Therefore, the interaction network between Hsp70s, GTPase Ran and importin proteins in rice might be involved in translocation of macromolecules. Shulga et al. (1996) stated that Hsp70 could act as a molecular chaperone to promote the formation and stability of the nuclear localization signal-containing complex during both targeting and translocation phases of nuclear transport [55].

### Sub-network B: Plant carbohydrate metabolism

The results of this study revealed that Hsp70s interacted with enolase (LOC_Os09g20820), fumaratehydratase (LOC_Os03g21950), malate dehydrogenase (LOC_Os07g43700, LOC_Os01g61380, LOC_Os05g49880) and citrate synthase (LOC_Os02g10070), which were constructed in sub-network B (Figure 9B). Most of these potential interactions have been partly validated by previous studies. *In vitro* studies indicated that Hsp70 might assist in transporting fumaratehydratase between the cytosol and mitochondria [56]. Furthermore, it has been reported that the Hsp70 complex significantly increased the spontaneous rate of refolding of denatured mitochondrial malate dehydrogenase [57]. Hsp70s have also been demonstrated to reduce the aggregation of citrate synthase under heat stress [58]. Recently, through co-immunoprecipitation (CoIP) assays, Luo et al. (2011) further confirmed that Hsp70 could directly interact with α-enolase [59].

Our results indicated that the expression levels of Hsp70s were positively and strongly correlated with that of enolase, fumaratehydratase, malate dehydrogenase and citrate synthase in response to abiotic stresses (Additional file 5: Figure S2; Additional file 1: Table S6), implying that Hsp70s might have essential functions in stimulating carbohydrate metabolism by regulating the activity of those key enzymes. In a metabolomics study, Kaplan et al. (2004) also found that carbohydrate metabolism was affected by heat shock in *Arabidopsis*[60]. The amount of pyruvate and oxaloacetate increased coordinately upon heat shock, while the fumarate and malate (oxaloacetate precursors) contents were similarly elevated, suggesting that the Embden-Meyerhof-Parnas (EMP) pathway and tricarboxylic acid cycle (TCA) cycle would be enhanced by abiotic stresses.

### Sub-network C: plant innate immunity

In this study, we found that Hsp70s might cooperate with members of the small GTPaseRac family (LOC_Os01g12900, LOC_Os02g02840, LOC_Os02g20850), Hsp90 (LOC_Os06g50300, LOC_Os08g39140), SKP1 (LOC_Os09g36830) and MAPK6 (LOC_Os06g06090), as shown in Figure 9C. Hsp70, Hsp90 and RAR1 have been documented as the components of Rac1 complex in rice, based on CoIP experiments [61]. Moreover, multiple lines of evidence have shown that Hsp70 was a negative regulator of ASK1/MAP3K, and overexpression of Hsp70 inhibited the MAPK signaling cascade, which was associated with apoptosis [62–64]. Consistent with previous studies, our results further illustrated that the expression level of Hsp70s was positively correlated with that of Rac, Hsp90 and SKP1, and negatively correlated with that of MAPK6 in response to abiotic stresses (Additional file 5: Figure S3; Additional file 1: Table S7). Furthermore, in addition to Rac (PF00071 and PS00017, PS51420), MAPK6 (PF00069 and PS50011, PS00108, PS00107, PS01351) also contained potential binding sites for Hsp70s.

Previous reports have shown that Hsp90 and two co-chaperone-like molecules, RAR1 and SGT1, performed a key role in effector-triggered immunity (ETI), the second line of the plant defense system [61, 65, 66]. Additionally, *in vitro* studies have indicated that SGT1 can interact with SKP1 and link it to the Hsp90 co-chaperone complexes [67]. Further research found that the SKP1-CULLIN1-F-box (SCF) complex regulated the stability of resistance (R) proteins [68], suggesting that SKP1 might also be involved in the ETI response. In addition, the small GTPase Rac could function as a critical switch downstream of two types of innate immunity: PAMP-triggered immunity (PTI) and effector-triggered immunity (ETI) [66]. This finding was recently supported by Jung et al. (2013). They found that the OsctHsp70-1 had a functional association with Ras/Raf-mediated MAPK kinase cascades [14].

### Sub-network D: photosystem II repair

Sub-network D showed that Hsp70s might interact with FtsH families (LOC_Os06g51029, LOC_Os01g62500 and LOC_Os01g43150) (Figure 9D). Indeed, this interaction has been previously confirmed by Shen and colleagues [69]. In this study, we found that there was a close positive correlation (PCC > 0.90) between the expression of Hsp70s and FtsH families in rice subjected to abiotic stresses (Additional file 5: Figure S4; Additional file 1: Table S8). The AAA-protein family signatures (PF00004, PS00674) of FtsH proteins were identified as potential target sites for Hsp70s. Previous showed that FtsH family members played an important role in the D1 repair cycle of PSII [70–72]. Using native gel electrophoresis, Yokthongwattana et al. (2001) revealed that Hsp70s could form a complex with intact D1 protein and also with D2 and CP47 [73], suggesting Hsp70s have a function in the photosystem II (PSII) repair cycle.

### Sub-network E: protein kinase activities

In this study, we found that nearly 46% of the Hsp70 interactors (197 out of 430) contained protein kinase domains, including protein kinase C (PKC), protein kinase A (PKA), apoptosis signal-regulating kinase/mitogen-activated protein kinase kinasekinase (ASK/MAP3K), mitogen-activated protein kinase kinase (MAP2K), mitogen-activated protein kinase (MAPK), cyclin-dependent kinase (CDK), Ca^2+^-dependent protein kinase (CDPK), CBL-interacting protein kinase (CIPK), osmotic stress/abscisic acid-activated protein kinase (SAPK) and wall-associated kinase (WAK) family members. Furthermore, our results showed that the expression level of approximately 81% of those protein kinases (159 out of 197) had a strong negative correlation (PCC < −0.90) with that of Hsp70s. This was consistent with previous studies. Hsp70s were reported to directly interact with PKC, ASK/MAP3K and CDK [63, 74, 75], and inhibit the activities of jun amino-terminal kinase (JNK), ASK/MAP3K, MAPK and CDK [3, 63, 74–76]. Ding et al. (1998) have shown that overexpression of Hsp70 significantly suppressed the enzymatic activities of PKA and PKC [77]. Therefore, it is likely that Hsp70s indiscriminately down-regulate the activity of various protein kinases.

## Conclusions

By integrating orthology and functional association data, we identified 27 Hsps in rice, including 12 sHsps, 6 Hsp70s, 3 Hsp60s, 3 Hsp90s and 3 Hsp100/ClpBs. Then, using Hsp70s as a case study, we identified 430 interactors of Hsp70s in rice by combining interolog- and expression profile-based methods. According to the interactors of Hsp70s, we investigated the potential binding sites of Hsp70s, and analyzed the interacting network of Hsp70s in rice. Finally, we constructed two online databases and one tool, which could be accessed at http://bioinformatics.fafu.edu.cn/.

## Methods

### Data sources

#### Rice sequence data

Rice proteome sequences were obtained from the Rice Genome Annotation Project (RGAP version 6.0; http://rice.plantbiology.msu.edu/) [78].

#### Yeast interaction data

Eight hundred and thirty-seven experimentally verified protein-protein interaction (PPI) pairs related to Hsps in yeast (Additional file 1: Table S2) were manually selected from the Database of Interaction Proteins (DIPs version 20101010; http://dip.doe-mbi.ucla.edu/dip/).

#### Microarray dataset

Gene expression data for rice subjected to drought, salt, cold or heat treatments were downloaded from GEO (accession number GSE6901 for drought, salt and cold treatments, and GSE14275 for heat treatment). All data were obtained using the same microarray platform (Affymetrix GeneChip Rice Genome Array; platform accession number GPL2025) and rice seedling samples (Table 1).

### Microarray analysis

#### Preprocessing of microarray data

The impute package (version 1.22.0) [31, 79] in Bioconductor [80] was used to estimate missing expression data. In addition, probe-sets, whose expression value was absent in GSE6901 or GSE14275, were filtered out. Furthermore, a robust scatterplot smoother (LOWESS) [81] in R software (version 2.10.1) [82] was used to perform intensity-dependent within-slide normalization [83]. The Limma package (version 3.2.0) was implemented to scale multiple-slide normalization [84].

#### Heat-responsive probe-sets detection

Boxplot [32, 33] in R was implemented to identify heat-responsive (HR) probe-sets. Probe-sets with M-values (log ratios) located beyond the upper or lower fence of the boxplot were considered as HR gene probe-sets.

#### Estimation of the global median absolute value of Pearson Correlation Coefficient (PCC)

The bootstrap method [34] was used to evaluate the median absolute value of PCCs between the expression levels of any two probe-sets among GeneChips. First, 10,000 non-redundant probe pairs were randomly selected, and the absolute PCC between each pair was computed. Based on these 10,000 PCC values, 100,000 bootstrap samples were built by sampling with replacement to measure the 95% confidence interval of the global median absolute value of PCC.

### Identification of rice Hsps

Rice candidate Hsps were selected from the Uniprot database. These sequences satisfied the following criteria: (1) they possessed the conserved domains of Hsps (Additional file 1: Table S1); (2) they were functionally annotated as Hsps or involved in similar biological processes; (3) the sequence length was in agreement with the molecular mass of different Hsp family members; (4) Evidence at RNA or protein expression level; and (5) they were identified as Hsps in the MSU Rice Genome Annotation Project. After that, their corresponding Affymetrix IDs were retrieved from Ricechip.org (http://www.ricechip.org/). R software was used to calculate the PCC values between expression data of each candidate Hsp and HR gene probe-set.

### Prediction of proteins interacting with Hsp70s in rice

#### Interolog approach

For each experimentally verified PPI of Hsps, the pairwise amino acid sequence was locally run through BLASTP (version 2.2.23+) [85] against the entire rice proteome in an effort to identify orthologs in rice. The E-value cutoff, identity and alignment coverage were set at 10^−10^, 30% and 40%, respectively. Based on the core principle of interolog [19, 21], corresponding orthologous pairs in rice were predicted to interact with each other. Briefly, if two interacting proteins, A and B, in yeast had the corresponding orthologs A’ and B’ in rice, respectively, A’ might interact with B’.

#### Expression profile-based method

For each PPI predicted by interolog, we determined the absolute value of PCC between the corresponding gene expression data. R software was used to calculate the PCC values. Generally, the PCC values ranged from −1 to 1. A value of 1 indicated that the gene expression level of protein A would increase as that of protein B increased. In contrast, a value of −1 implied that the gene expression level of protein A would decrease as that of protein B increased. A value of 0 implied that there was no linear correlation between the expressions of these two genes. If the absolute value was less than 0.90, the PPI was filtered out.In addition, t-test was utilized to evaluate whether the paired PCC value was significantly greater or less than 0.

### Assessment of PPIs of Hsp70s in rice

#### Protein localization method

Subcellular localization information of proteins in rice was obtained from WoLF PSORT [39]. In addition, 1,000 randomized networks, in which the interacting partners of Hsp70s were randomly replaced by other proteins containing meaningful subcellular localization annotations in the rice proteome, were used as a control. The above process was repeated 1,000 times.

#### Function similarity method

The GO annotations of proteins in rice were downloaded from agriGO (http://bioinfo.cau.edu.cn/agriGO/download.php) [86]. Furthermore, 1,000 randomized repeats of Hsp70 interactors were generated. The predicted interactors of Hsp70s were randomly replaced by other proteins possessing GO annotations in the rice proteome. The above procedure was repeated 1,000 times.

### Identification of binding sites of Hsp70s in rice

#### Non-interactors dataset

Non-interactors of Hsp70s were used as negative controls. These proteins were collected from the rice proteome, and satisfied the following conditions: first, they could not interact with Hsp70s, based on the interolog prediction; and second, the absolute PCC value between the expression level of the non-interactor and that of any Hsp70 should be less than 0.40.

#### Domain assignment

The domain information of rice proteins was obtained from Pfam (http://pfam.sanger.ac.uk/) [41]. Because of the large number of sequences, we ran the PfamScan program (version 091007) [41] and HMMER package (version 3.0b3) [87] locally. Rice protein sequences were searched against Pfam-A domains in PfamScan databases (version 24.0) with an E-value cutoff of 0.0001.

#### Motif assignment

The motif annotations of proteins in rice were acquired from PROSITE (http://prosite.expasy.org/) [45]. The ScanProsite tool [44] was downloaded and applied locally to scan protein sequences against the PROSITE database (version 20.67).

#### Fisher’s exact test

A one-tailed Fisher’s exact test was used to detect the over-represented domains and motifs among the Hsp70s interactors in rice compared with the negative interactors. For each domain or motif annotation, a 2 × 2 contingency table was constructed, as shown in Additional file 1: Table S4. Then, R software was used to calculate the *p*-value to measure the significance level.

#### Multiple testing

To limit the false-positive error rate associated with multiple statistical tests, R software was further used to alter each *p*-value into the corresponding adjusted *p*-value based on the BH method [43]. Ultimately, the adjusted *p*-value was used to determine the potential binding sites. A cutoff value of 0.05 was used in this work.

### Hsp70 network in rice

#### GO enrichment

The GO information of the predicted Hsp70 interactors in rice was obtained from agriGO (http://bioinfo.cau.edu.cn/agriGO/). For each GO term, all parent nodes were retrieved according to the archive of the GO database, and the minimum distance from the root (depth) was determined. Only terms beyond the fourth depth were considered. After that, fisher’s exact test was conducted to reveal the over-represented GO terms in the opposite dataset, and the BH method was used to control the false discovery rate (FDR). The Hsp70 network was generated using Cytoscape (http://www.cytoscape.org/)[88].

### Construction of tools and the rice Hsps database

The web tools and rice Hsps database were constructed on a LAMP (Linux, Apache, MySQL and PHP) platform. RGEP visualization was developed using two types of open source software, Open Flash Chart (http://teethgrinder.co.uk/open-flash-chart/) and Google Chart Tools (https://developers.google.com/chart/).

## Electronic supplementary material

Additional file 1: Table S1: Domains for heat shock protein query in Uniprot database. **Table S2.** Number of PPIs related to Hsps in yeast collected from DIP. **Table S3.** Number ofpredicted protein-protein interaction related to rice Hspsby using interolog method. **Table S4.** 2 × 2 contingency table for Fisher’s exact test. **Table S5.** PCC between Hsp70s and Ran, importin proteins respectively. **Table S6.** PCC between Hsp70s and fumaratehydratase, malate dehydrogenase and citrate synthase respectively. **Table S7.** PCC between Hsp70s and Racs, Hsp90, SKP1 respectively. **Table S8.** PCC between Hsp70s and FtsH proteins. (DOC 78 KB)

Additional file 2: **Supplemental Data 1A.** Predicted PPIs ofHsp70s in rice based on the interolog and gene expression-based methods. **Supplemental Data 1B.** Predicted interactors of Hsp70s. (XLS 297 KB)

Additional file 3: **Supplemental Data 2A.** Domains overrepresented among interactors of Hsp70s. **Supplemental Data 2B.** Motifs overrepresented among interactors of Hsp70s. (XLS 28 KB)

Additional file 4: **Supplemental Data 3A.** Enriched GO terms among interactors with expression levels positively correlated with Hsp70s. **Supplemental Data 3B.** Enriched GO terms among interactorswith expression levels negatively correlated with Hsp70s. (XLS 32 KB)

Additional file 5: Figure S1: Gene expression profile of Hsp70s, Ran and importin proteins in response to abiotic stresses. **Figure S2.** Gene expression profile of Hsp70s, enolase, fumaratehydratase, malate dehydrogenase and citrate synthase in response to abiotic stresses. **Figure S3.** Gene expression profile of Hsp70s, Racs, Hsp90s, SKP1 in response to abiotic stresses. **Figure S4.** Gene expression profile of Hsp70s and FtsH proteins in response to abiotic stresses. (DOC 80 KB)
