# Supplementary material for: Genome-wide identification of heat shock proteins (Hsps) and Hsp interactors in rice: Hsp70s as a case study
Source: BMC Genomics. 2014 May 7;15(1):344. doi: 10.1186/1471-2164-15-344 (PMC4035072; doi:10.1186/1471-2164-15-344)
Supplement: Supplementary file 1 — Additional file 1: Table S1: Domains for heat shock protein query in Uniprot database. Table S2. Number of PPIs related to Hsps in yeast collected from DIP. Table S3. Number ofpredicted protein-protein interaction related to rice Hspsby using interolog method. Table S4. 2 × 2 contingency table for Fisher’s exact test. Table S5. PCC between Hsp70s and Ran, importin proteins respectively. Table S6. PCC between Hsp70s and fumaratehydratase, malate dehydrogenase and citrate synthase respectively. Table S7. PCC between Hsp70s and Racs, Hsp90, SKP1 respectively. Table S8. PCC between Hsp70s and FtsH proteins. (DOC 78 KB) [file 12864_2013_6029_MOESM1_ESM.doc]

**Table S1.** **Domains for heat shock protein query in Uniprot database**

| **Family** | **Pfam** |
| --- | --- |
| Small Hsp | PF00011 |
| Hsp60 | PF00118 |
| Hsp70 | PF00012 |
| Hsp90 | PF00183 |
| Hsp100/ClpB | PF00004;PF07724;PF02861;PF10431 |

**Table S2**. Number of PPIs related to Hsps in yeast

| **Uniprot ID** | **DIP ID** | **Name** | **Family** | **Interactions** |
| --- | --- | --- | --- | --- |
| P31539 | DIP-2252N | Hsp104 | Hsp100/ClpB | 20 |
| P15108 | DIP-1524N | Hsc82 | Hsp90 | 52 |
| P02829 | DIP-2262N | Hsp82 | Hsp90 | 65 |
| P09435 | DIP-2266N | Hsp73/SSA3 | Hsp70 | 33 |
| P10591 | DIP-2253N | Hsp71/SSA1 | Hsp70 | 15 |
| P11484 | DIP-2254N | Hsp75/SSB1 | Hsp70 | 48 |
| P22202 | DIP-3916N | Hsp74/SSA4 | Hsp70 | 21 |
| P32589 | DIP-6645N | Hsp7F/SSE1 | Hsp70 | 5 |
| P32590 | DIP-4862N | Hsp79/SSE2 | Hsp70 | 14 |
| Q05931 | DIP-6407N | Hsp7Q/SSQ1/SSC2 | Hsp70 | 1 |
| P12398 | DIP-411N | Hsp70/SSC1 | Hsp70 | 175 |
| P10592 | DIP-2265N | Hsp72/SSA2 | Hsp70 | 13 |
| P39987 | DIP-6541N | Hsp7E/SSC3/ECM10 | Hsp70 | 18 |
| P19882 | DIP-7648N | Hsp60 | Hsp60 | 168 |
| P15992 | DIP-3988N | Hsp26 | sHsp | 113 |
| P22943 | DIP-6408N | Hsp12 | sHsp | 4 |
| P25619 | DIP-4586N | Hsp30 | sHsp | 7 |
| Q12329 | DIP-2515N | Hsp42 | sHsp | 61 |
| P38910 | DIP-1483N | Hsp10 | sHsp | 9 |
| Total* |  |  |  | 837 |

* Including 5 PPIs between heat shock proteins.

**Table S3. Number of predicted protein-protein interaction related to rice Hsps using interolog method.**

| **Family** | **Numbers** | **Interaction** |
| --- | --- | --- |
| sHsp | 4/12 | 1383 |
| Hsp60 | 3/3 | 1854 |
| Hsp70 | 6/6 | 4091 |
| Hsp90 | 3/3 | 1398 |
| Hsp100 | 3/3 | 432 |
| Total* | 19/27 | 9132 |

* Including 26 PPIs between heat shock proteins

**Table S4. 2×2 contingency table for fisher exact test. a and c are the number of certain domain or motif annotation presented in the positive samples and negative samples respectively. While the frequency of not-existence in the positive samples and negative samples were** represented by b and d respectively.

|  | **Existence** | **Not-existence** | **Total** |
| --- | --- | --- | --- |
| **Positive samples** | a | b | a+b |
| **Negative samples** | c | c | c+d |
| **Total** | a+c | b+d | a+b+c+d |

**Table S5. Pearson correlation coefficient between Hsp70s and Ran, Importin** proteins.

| **P C C** | **LOC_Os03g11910**  **Hsp70-A** | **LOC_Os12g14070**  **Hsp70-B** |
| --- | --- | --- |
| LOC_Os01g42530  small GTPase Ran | 0.99804 | 0.97204 |
| LOC_Os01g14950  Importin subunit alpha-1a | 0.97722 | 0.89574 |
| LOC_Os05g06350  Importin subunit alpha-1b | 0.98342 | 0.89499 |
| LOC_Os05g28510  importin subunit beta | 0.76602 | 0.9033 |

**Table S6. Pearson correlation coefficient between Hsp70s and fumarate hydratase, malate dehydrogenase and citrate synthase**.

| **P C C** | **LOC_Os03g11910**  **Hsp70-A** | **LOC_Os12g14070**  **Hsp70-B** |
| --- | --- | --- |
| LOC_Os09g20820  enolase | 0.90691 | 0.93461 |
| LOC_Os03g21950  fumarate hydratase | 0.96637 | 0.91917 |
| LOC_Os07g43700  malate dehydrogenase | 0.93241 | 0.79622 |
| LOC_Os01g61380  malate dehydrogenase | 0.99994 | 0.95995 |
| LOC_Os05g49880  malate dehydrogenase | 0.9637 | 0.99704 |
| LOC_Os02g10070  citrate synthase | 0.93908 | 0.80509 |

**Table S7.** **Pearson correlation coefficient between Hsp70s and OsRacs, Hsp90, SKP1**

| **P C C** | **LOC_Os03g11910**  **Hsp70-A** | **LOC_Os12g14070**  **Hsp70-B** | **LOC_Os01g08560**  **Hsp70-C** |
| --- | --- | --- | --- |
| LOC_Os01g12900  OsRac1 | 0.9125 | 0.9734 | 0.4488 |
| LOC_Os02g02840  OsRac6 | 0.9640 | 0.9862 | 0.6509 |
| LOC_Os02g20850  OsRac7 | 0.8453 | 0.7178 | 0.9463 |
| LOC_Os06g50300  Hsp90 | 0.9440 | 0.8766 | 0.8632 |
| LOC_Os08g39140  Hsp90 | 0.5422 | 0.2904 | 0.9464 |
| LOC_Os09g36830  SKP1 | 0.9969 | 0.9620 | 0.7765 |
| LOC_Os06g06090  OsMAPK6 | -0.8511 | -0.9589 | -0.3252 |

**Table S8.** **Pearson correlation coefficient between Hsp70s and OsFtsH proteins**

| **P C C** | **LOC_Os03g11910**  **Hsp70-A** | **LOC_Os05g38530**  **Hsp70-D** |
| --- | --- | --- |
| LOC_Os06g51029  OsFtsH1 | 0.63442 | 0.99547 |
| LOC_Os01g62500  OsFtsH3 | 0.93905 | 0.83459 |
| LOC_Os01g43150  OsFtsH9 | 0.93045 | 0.52953 |
