# Supplementary material for: Genome-wide identification of heat shock proteins (Hsps) and Hsp interactors in rice: Hsp70s as a case study
Source: BMC Genomics. 2014 May 7;15(1):344. doi: 10.1186/1471-2164-15-344 (PMC4035072; doi:10.1186/1471-2164-15-344)
Supplement: Supplementary file 5 — Additional file 5: Figure S1: Gene expression profile of Hsp70s, Ran and importin proteins in response to abiotic stresses. Figure S2. Gene expression profile of Hsp70s, enolase, fumaratehydratase, malate dehydrogenase and citrate synthase in response to abiotic stresses. Figure S3. Gene expression profile of Hsp70s, Racs, Hsp90s, SKP1 in response to abiotic stresses. Figure S4. Gene expression profile of Hsp70s and FtsH proteins in response to abiotic stresses. (DOC 80 KB) [file 12864_2013_6029_MOESM5_ESM.doc]

**
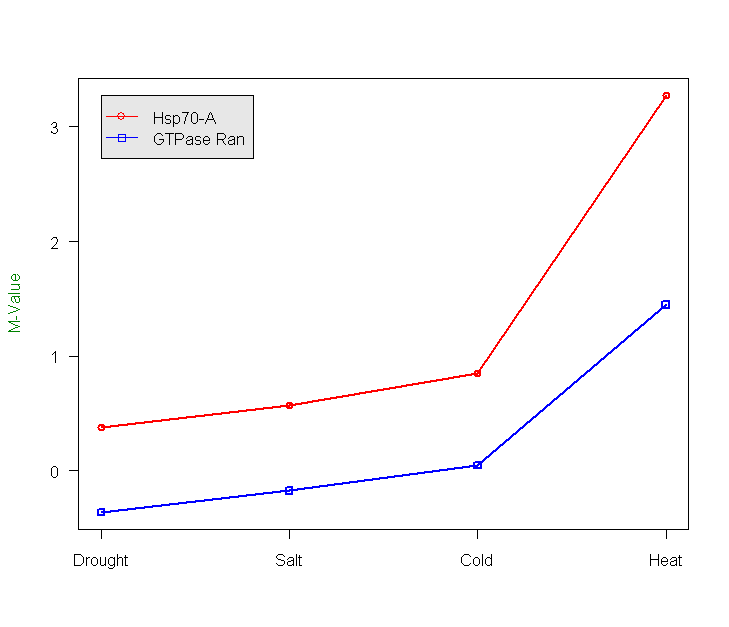

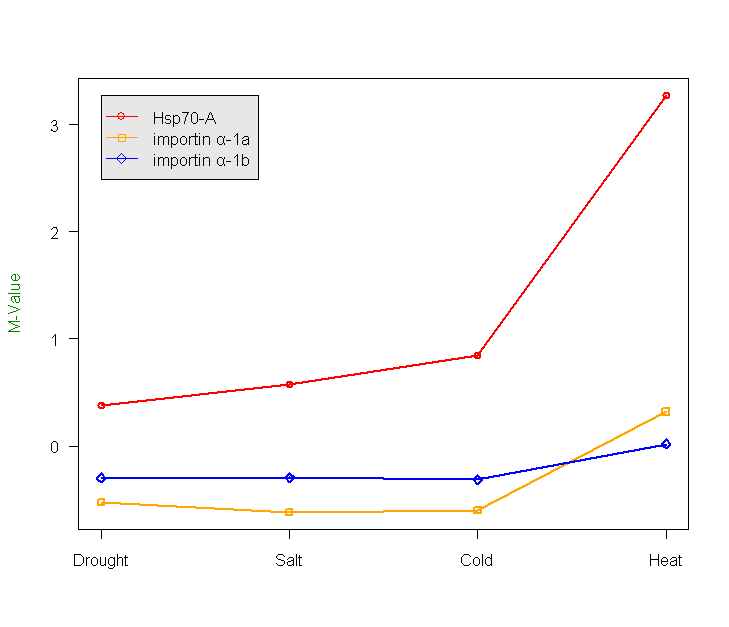
**

A

B

**
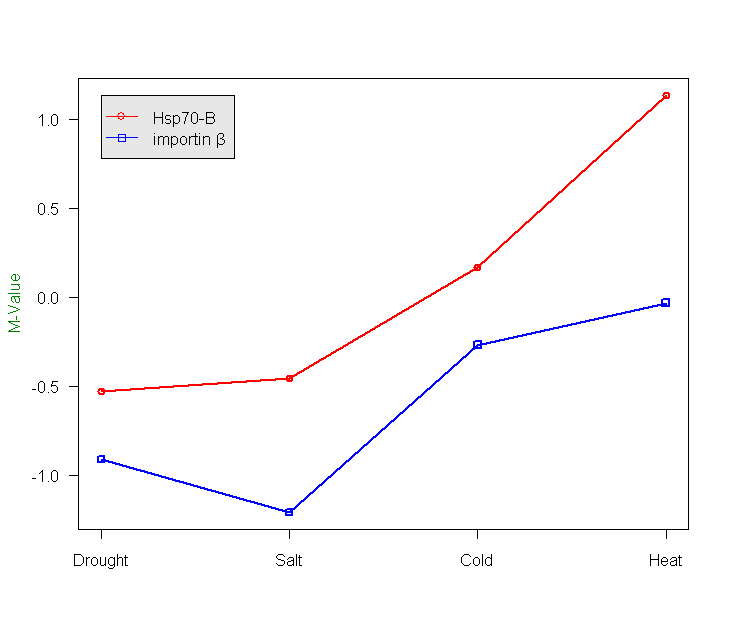
**

C

**Figure S1. Gene expression profile of Hsp70s, Ran and Importin proteins in response to abiotic stresses**

**
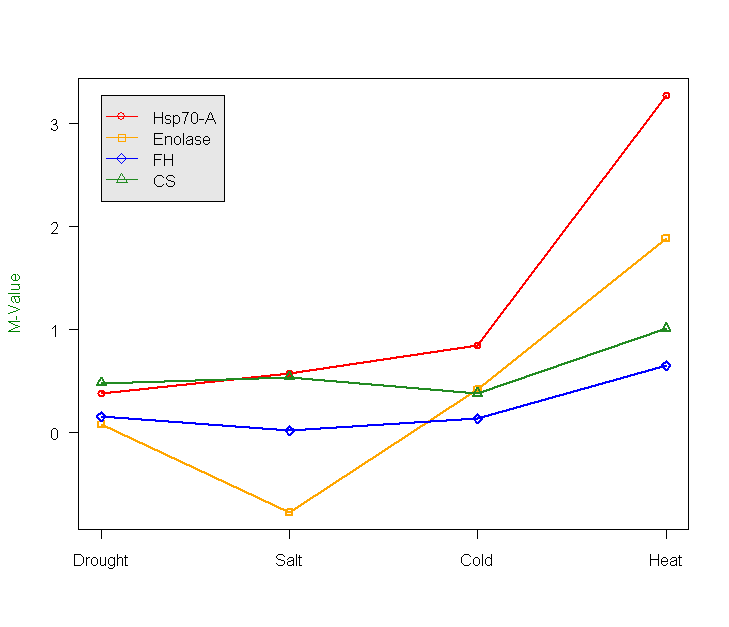
**

A

**
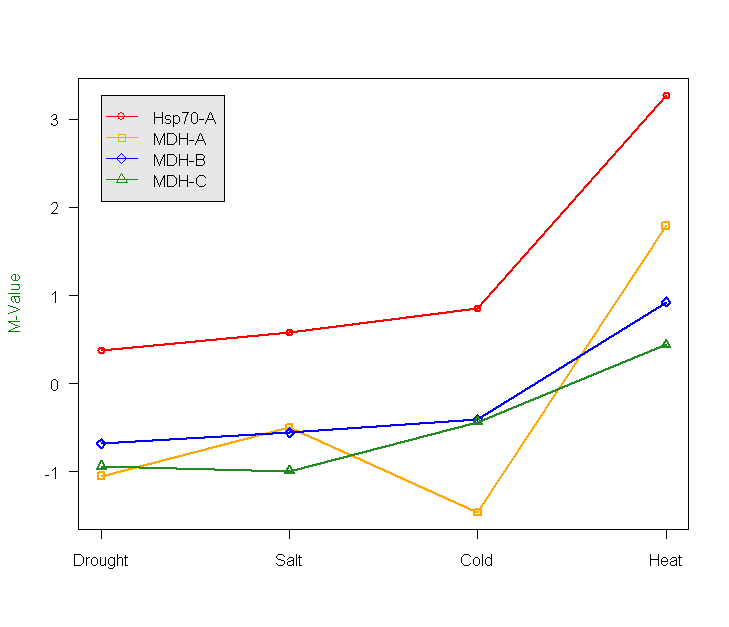
**

B

**Figure S2. Gene expression profile of Hsp70, enolase, fumarate hydratase, malate dehydrogenase and citrate synthase in response to abiotic stresses**

**
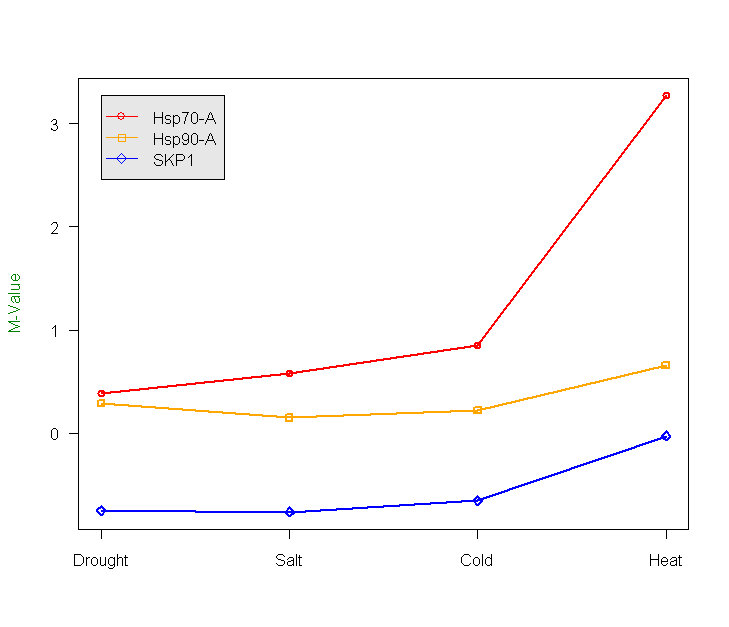

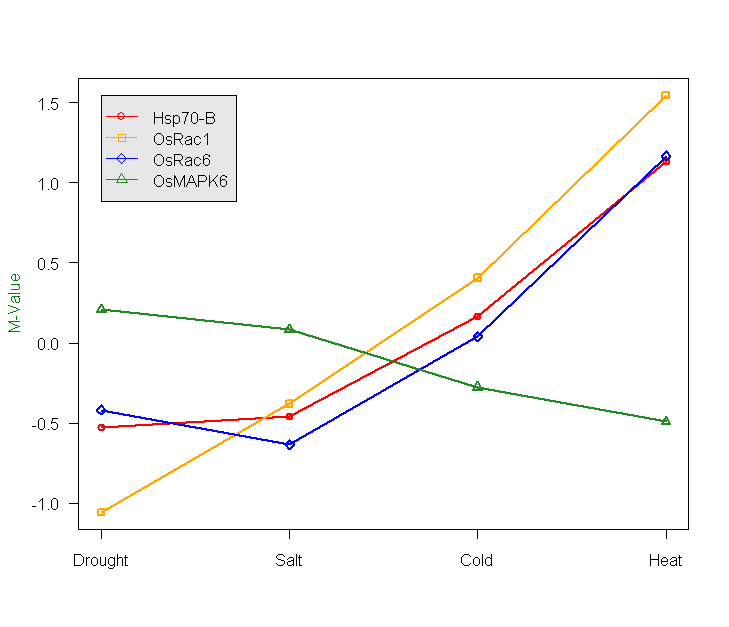
**

A

B

**
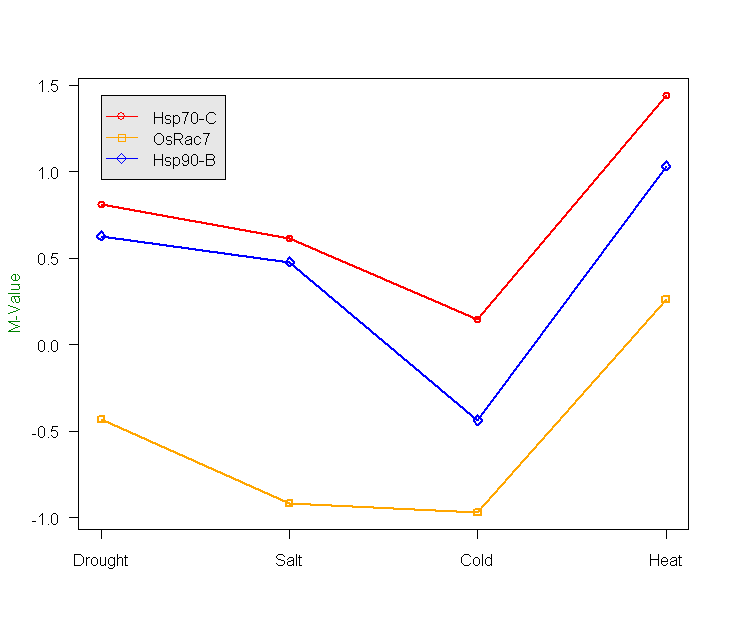
**

C

**Figure S3. Gene expression profile of Hsp70s, OsRacs, Hsp90s, SKP1 in response to abiotic stresses**

**
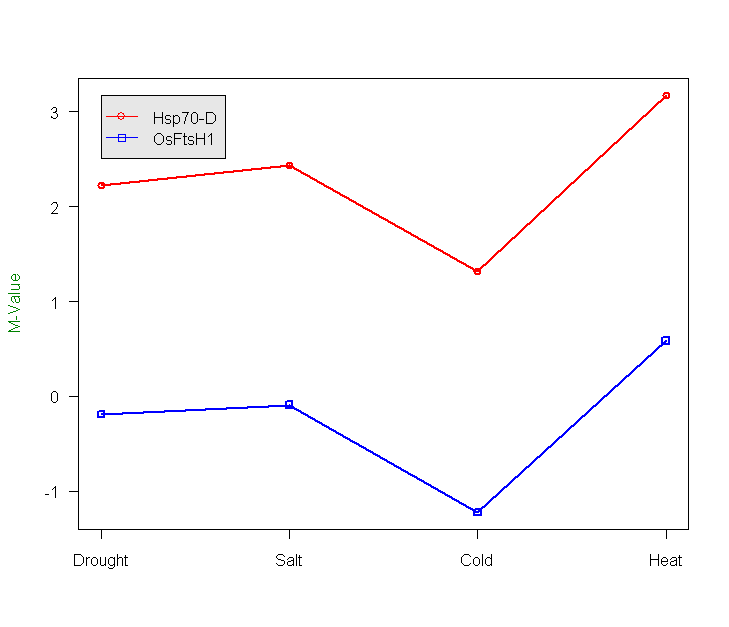
**

A

**
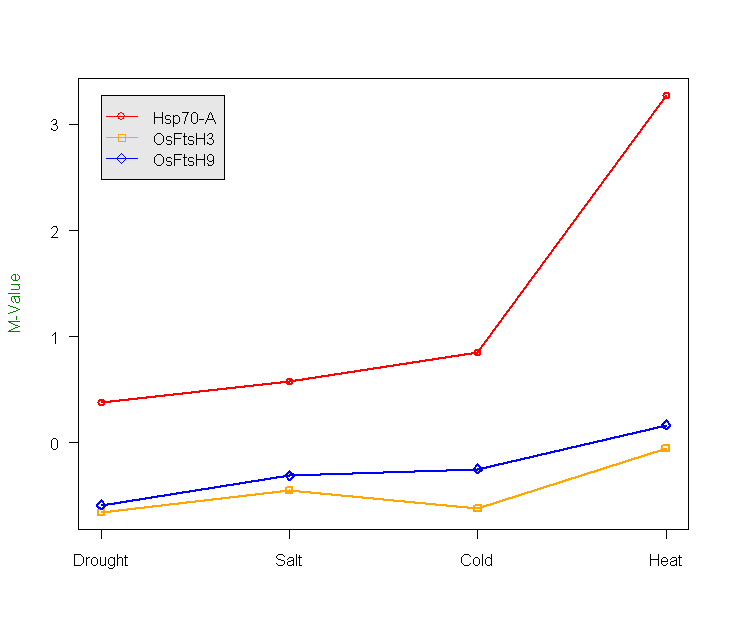
**

B

**Figure S4. Gene expression profile of Hsp70s and OsFtsH proteins**
